# Genetic Profile and Clinical Implications of Hepatoblastoma and Neuroblastoma Coexistence in a Child

**DOI:** 10.3389/fonc.2019.00230

**Published:** 2019-04-04

**Authors:** Szymon Skoczen, Konrad Stepien, Marta Krzysztofik, Teresa Luszawska, Malgorzata Hnatko-Kolacz, Michal Korostynski, Marcin Piechota, Katarzyna Kolanek, Lukasz Wyrobek, Katarzyna Wysocka, Wojciech Gorecki, Walentyna Balwierz

**Affiliations:** ^1^Department of Oncology and Hematology, University Children's Hospital, Krakow, Poland; ^2^Department of Pediatric Oncology and Hematology, Institute of Pediatrics, Jagiellonian University Medical College, Krakow, Poland; ^3^Student Scientific Group of Pediatric Oncology and Hematology, Jagiellonian University Medical College, Krakow, Poland; ^4^Department of Molecular Neuropharmacology, Institute of Pharmacology of Polish Academy of Sciences, Krakow, Poland; ^5^Intelliseq sp. z o.o., Krakow, Poland; ^6^Department of Radiology, University Children's Hospital, Krakow, Poland; ^7^Department of Pathology, University Children's Hospital, Krakow, Poland; ^8^Department of Pediatric Surgery, Institute of Pediatrics, Jagiellonian University Medical College, Krakow, Poland

**Keywords:** malignant neoplasms, children, hepatoblastoma, neuroblastoma, mutation

## Abstract

The aim of the following case report is to provide a description of the coexistence of two independent tumors in a child. A 9-month-old male was referred to Department of Pediatric Oncology and Hematology with hepatic tumor present on ultrasound imaging and symptoms of enlarged abdominal circumference. Physical examination revealed a palpable epigastric mass and the imaging techniques showed a tumor of the left hepatic lobe measuring 11 × 6.5 × 8.9 cm with pancreas infiltration, distant metastases in both lungs and abnormal lesion in the left adrenal gland. Basing on histopathological examination, after a core-needle biopsy, hepatoblastoma (HBL) (mixed epithelial-mesenchymal subtype) was diagnosed. The α-fetoprotein level was 112 993 ng/ml. Elevated values of normetanephrine, 3-methoxytyramine as well as neuron-specific enolase were observed. Due to the clinical picture and diagnosis, the patient was qualified to preoperative chemotherapy according to the SIOPEL-3 protocol, followed by SIOPEL-4 protocol for the high-risk patients. After undergoing preoperative chemotherapy, imaging tests revealed regression of hepatic tumor and no focal pulmonary masses, while regression of adrenal gland mass was not completed. The patient was qualified for left hemihepatectomy with left adrenalectomy. Histopathological examination of liver specimen confirmed the HBL diagnosis. However, in left adrenal gland and paraaortic lymph nodes the residual neuroblastoma (NBL) cells were detected. Whole exome sequencing (WES) was utilized to identify disease-associated germline mutations. WES revealed a novel germline insertion variant in *TWIST1* (p.Gly86dup), along with the potentially pathogenic non-synonymous variants in *NF1* (p.Val2511Ile), *RAF1* (p.Leu445Arg), and *WHSC1* (p.Ser4Asn) genes. Currently, 6 months after completion of treatment according to the SIOPEL-4 protocol, the patient is in good general condition, without any signs, and symptoms of relapse of both neoplasms. The coexistence of two different primary childhood malignancies is rarely seen. So far, only one case of synchronous HBL and NBL has been reported. However, for the first time therapeutic process was successful. A specific signature of rare germline mutations can be proposed as a predisposing factor to synchronous HBL and NBL occurrence.

## Introduction

Sporadic or multifactorial etiology is considered as a cause in most cases of cancer development in children. However, as has been shown, up to 10% of cancer cases that occur in children are related to known predisposing genetic syndromes (1). For instance, Beckwith-Wiedemann syndrome, Familial Adenomatous Polyposis, neurofibromatosis type 1 and Li-Fraumeni syndrome are disorders with increased tumor risk. An accurate molecular diagnosis gives a possibility to implement appropriate primary prevention or to detect developing cancer at an early stage by regular screening. In some cases, especially in the absence of a significant family history and characteristic dysmorphic features, the developing cancer disease may indicate the undiagnosed genetic condition. According to data from the literature, the type of diagnosed cancer accounts for 18% of the reasons for referring the child to further genetic testing (2). High-throughput methods such as next-generation sequencing (NGS) allow for an accurate and comprehensive genetic analysis in a relatively short time.

The presence of significant genetic disorders is extremely suspected in the rare coexistence of two independent solid tumors. Both hepatoblastoma (HBL), the most common primary malignant neoplasm of the liver in children and neuroblastoma (NBL), the most frequent extracranial solid tumor, belong to the same group of tumors consisting of immature cells which often resemble individual stages of embryonic organs development. The incidence of HBL according to US registers is 1.6 cases per million, while NBL−10.54 cases per million (3). Because of the low occurrence rates, accidental simultaneous development of two mentioned tumors in the same patient is very unlikely.

To our knowledge, in the available literature only one case of the coexistence of HBL and NBL in a child has been already described (4).

## Background

We report a case of a 9-month-old male referred to Department of Pediatric Oncology and Hematology with suspicion of hepatic tumor measuring 12.5 × 7 cm on ultrasound imaging. His medical history was remarkable for enlarged abdominal circumference lasting for 2 months, with no abdominal pain or gastrointestinal disorders. Physical examination revealed an extensive palpable epigastric mass. All kinds of dysmorphic features were excluded.

**Figure 1:**
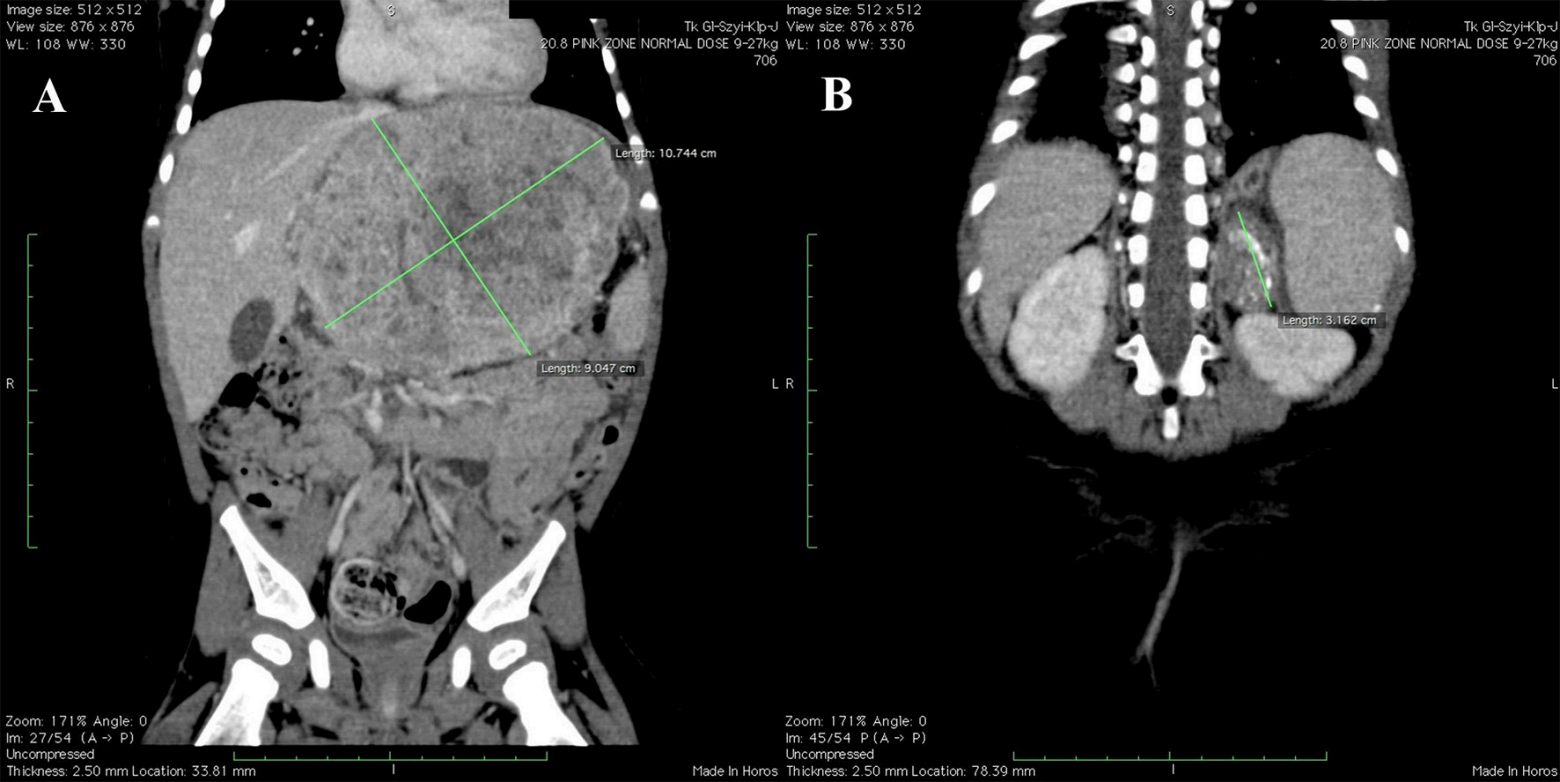
Initial CT scans. **(A)** Hepatoblastoma of the left hepatic lobe measuring 11 × 6.5 × 8.9 cm. **(B)** Neuroblastoma of the left adrenal gland measuring 2.3 × 1.6 × 3.6 cm.

**Figure 2:**
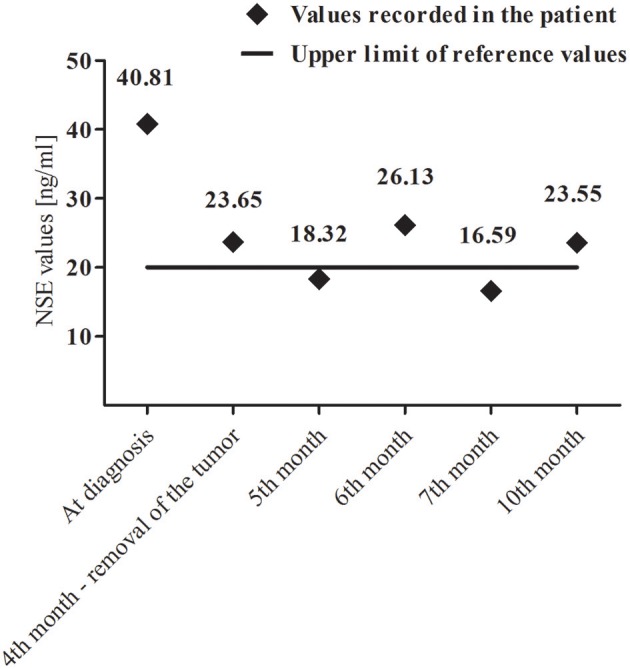
Variability of neuron-specific enolase (NSE) levels during and after combined-modality therapy.

**Figure 3:**
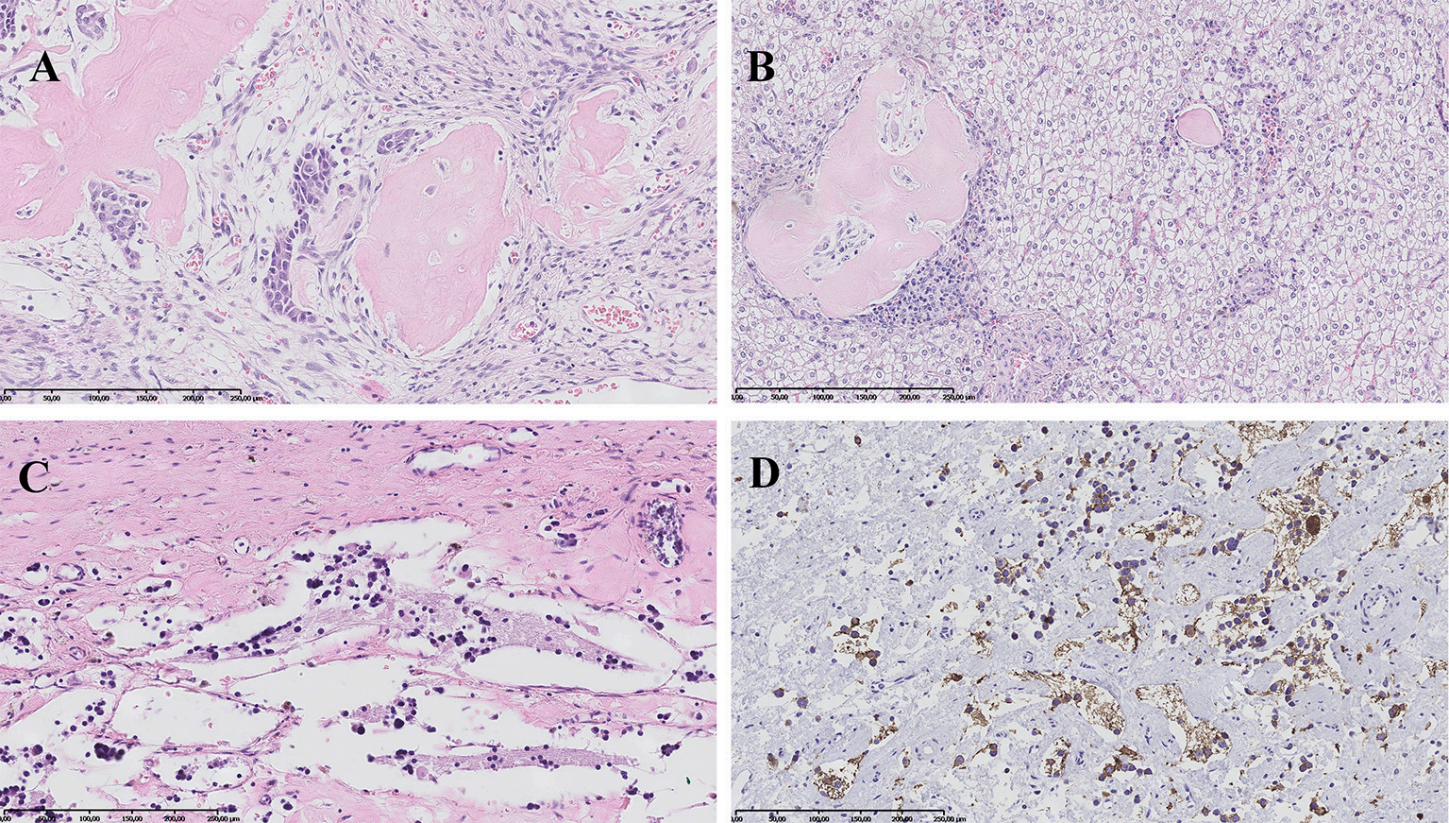
Histopathological images. **(A)** Hepatoblastoma, mixed epithelial-mesenchymal subtype: areas of embryonal epithelial type cells, primitive mesenchyme and osteoid formation. HE, magnification 200x. **(B)** Hepatoblastoma, mixed epithelial-mesenchymal subtype: fetal epithelial type cells with focal extramedullary hematopoesis and mesenchymal component represented by osteoid formation. HE, magnification 200x. **(C)** Neuroblastoma, Schwannian stroma—poor, differentiating subtype: scant differentiating neuroblasts and neuropil in the background. HE, magnification 200x. **(D)** Neuroblastoma, Schwannian stroma—poor, differentiating subtype: positive immunohistochemical reaction for synaptophysin in tumor cells cytoplasm and neuropil. Magnification 200x.

Computed tomography (CT) imaging was performed as a part of routine surveillance and showed: (1) tumor of the left hepatic lobe measuring 11 × 6.5 × 8.9 cm, infiltrating pancreas and adhering to falciform ligament of the liver, neck of gallbladder, main trunk of portal vein, common hepatic artery and inferior vena cava [PRETEXT III: V2, P1(2), E+, M+] (Figure 1A); (2) hypodense abnormal lesion in the left adrenal gland with calcifications and no contrast enhancement, measuring 2.3 × 1.6 × 3.6 cm (Figure 1B); (3) enlarged left paraaortic lymph nodes up to 1.4 cm with single calcifications; (4) distant peripheral and subpleural metastatic lesions in both lungs.

A core-needle biopsy of segment IV and V of the liver revealed HBL (mixed epithelial-mesenchymal subtype). The initial α-fetoprotein (AFP) level was 112 993 ng/ml (age/sex-adjusted reference ranges: 7–53 ng/ml). Patient presented no viral hepatitis infections. Due to the presence of abnormal mass in left adrenal gland, urinary excretion of catecholamines and their metabolites as well as serum neuron-specific enolase (NSE) levels were measured. Increased values of normetanephrine (1.32 μg/mg creatinine, [0.27–1.11]), 3-methoxytyramine (1.31 μg/mg creatinine, [0.37–1.24]) and NSE [40.81 ng/ml, (4–20)] were noted. Fluctuations of NSE in the perioperative period are presented in Figure 2.

Due to the clinical picture and diagnosis, the patient was qualified for preoperative chemotherapy according to the SIOPEL-3 protocol (cisplatin, carboplatin, doxorubicin) (5). Therapy was complicated by anemia requiring leukocyte-reduced red blood cell transfusion. In the course of the treatment, diminished abdominal circumference, and decrease of serum AFP level to 24 465 ng/ml were observed. In 4th week of chemotherapy CT imaging was performed and revealed reduced tumor masses in liver (PRETEXT II: V1, E1), left adrenal gland and lungs. Further treatment was continued according to the SIOPEL-4 protocol (cisplatin, doxorubicin) (6). Anemia and leukopenia requiring the subsequent leukoreduced transfusion as well as bilateral symmetric high-frequency hearing loss detected by the otoacoustic emissions method have occurred during the therapy. Control CT performed after the completion of preoperative chemotherapy showed a decrease in hepatic tumor mass to 5.6 × 5.2 × 6.3 cm and a complete remission of focal lesions in the lungs. However, there was no further reduction of the hypodense lesion in the left adrenal gland (2.2 × 1.4 × 2.5 cm). Gradual decrease in AFP concentration to 191.7 ng/ml was also observed.

The patient was qualified for surgical treatment: left hemihepatectomy and left adrenalectomy. Elective surgical procedure was performed 4 months after the beginning of oncological treatment. The histopathological examination of the removed liver tumor confirmed the initial diagnosis of HBL (mixed epithelial-mesenchymal subtype) (Figures 3A,B). In turn, in the material from adrenal tumor residual remnants of NBL (Schwannian stroma—poor, differentiating subtype) (synaptophysin +, NSE +) were found in the regression stage (Figures 3C,D). Calcifications with ambiguous single cells were visualized also in the left paraaortic lymph nodes (synaptophysin +/−, NSE +/−). Furthermore, analysis of the tumor material with array comparative genomic hybridization (aCGH) showed lack of *MYCN* amplification.

In the early postoperative period, the bile leakage was observed without the formation of fluid collections. In the next days of hospitalization symptoms of transient gastrointestinal obstruction resolved and parenteral nutrition was gradually reduced. Wound healing proceeded properly.

Currently the patient is in good general condition, without any signs and symptoms of relapse of both neoplasms, 6 months after completion of treatment according to the SIOPEL-4 protocol for HR Super PLADO.

## Genetic Testing

Due to the clinical course of the disease and histopathologically confirmed coexistence of two independent solid tumors, genetic tests were carried out. Genomic DNA was extracted from the peripheral blood samples according to the standard protocols. Whole exome sequencing (WES) was performed on Illumina HiSeq X Ten platform, using sequencing libraries generated with Agilent SureSelect Human All Exon V6 Kit (60 Mb target size, covering 99% of exons regions from RefSeq, CCDS, HGMD_cds, and OMIM_cds databases) and TruSeq DNA Library Preparation Kit. The raw reads from FASTQ files were aligned to hs38DH reference genome using BWA MEM algorithm (version 0.7.15). Resulting BAM files were post-processed with Sentieon software implementing the equivalent GATK 3.5 Best Practices protocol and the GVCFs files were generated.

The personalized panel of genes was created based on Human Phenotype Ontology (HPO) (version: April 13, 2017). The panel consists of genes associated with the phenotype: “HP:0003006 Neuroblastoma”; “HP:0002884 Hepatoblastoma”, and “HP:0002664 Neoplasm” (646 genes total). In order to calculate the coverage of obtained genes across samples, the custom script was employed. SNVs (Single Nucleotide Variants) and INDELs (short INsertions and DELetions) were discovered via joint genotyping of GVCFs.

The resulting VCF was annotated and filtered using an analysis of designed workflow. After discarding variants with QUAL <30, remaining variants were annotated on variant and gene level. The variant level annotations consisted of: flags designating the presence of variant [Online Mendelian Inheritance in Man (OMIM), PubMed or Human Gene Mutation Database (HGMD) databases], names of diseases associated with a given variant and their clinical significance [ClinVar (version: April 4th, 2017) and MITOMAP (version: January 19th, 2017) databases] and their frequencies in 1000 Genomes, ExAC, gnomAD, MITOMAP and local database. Variants were also annotated with impact prediction scores, such as SnpEff annotations (version: 4.3i, database version: GRCh38.86), SIFT—deleteriousness score (version: February 2th, 2017), GERP and PhastCons—evolutionary conservation scores. Variant-level filtering removed variants more frequent than 1% in 1000 Genomes, ExAC, or MITOMAP populations and variants with SnpEff impact LOW or MODIFIER. Gene-level filtering retained variants located in genes present in the panel. All variants that have passed filters defined above were additionally inspected using Integrative Genomics Viewer (IGV) to detect and exclude from further analysis false-positive calls.

Among genes associated in HPO database with autosomal dominant model of disease inheritance, there were 4 potentially pathogenic variants identified. The rare non-synonymous variants in *RAF1* (p.Leu445Arg) and *WHSC1* (p.Ser4Asn) were found. Both mutations have high SIFT deleteriousness score (<0.11) and high evolutionary conservation (GERP >4.7 and PhastCons = 1). A novel insertion variant in neoplasm-related gene *TWIST1* (p.Gly86dup) was identified, however, insertion did not produce a translational frameshift and occurred in the poorly evolutionary conserved region. There were also two variants in neoplasm-related *NF1* gene, including ultra-rare non-synonymous variant (p.Val2511Ile) in a highly conserved region (GERP > 4.0 and PhastCons = 1) though with poor SIFT score (0.552) and synonymous likely benign variant (p.Lys1745 =). WES also detected known pathogenic variant in *MYOC* (p.Gln368^*^). The mutation was reported in ClinVar as pathogenic with autosomal dominant inheritance for primary open angle glaucoma. The obtained results of the analysis are presented in abbreviated form in Table 1.

**Table 1:** Selected potentially significant pathogenic genetic variants detected in the patient.

**Gene symbol**	**Position of sequence change**	**Variant parameters**	**Inheritance pattern**	**Classification**	**Selected diseases associated with the gene**
*NF1*	p.Val2511Ile	SIFT = 0.552 PhastCons = 1 GERP = 4.08	Autosomal dominant, somatic mutation	Potentially significant variant from dedicated panel	Neurofibromatosis type 1, juvenile myelomonocytic leukemia, Noonan syndrome
*RAF1*	p.Leu445Arg	SIFT = 0.098 PhastCons = 1 GERP = 4.71	Autosomal dominant	Potentially significant variant from dedicated panel	Neuroblastoma
*TWIST1*	p.Gly86dup	PhastCons = 0 GERP = −0.67	Autosomal dominant	Potentially significant variant from dedicated panel	craniosynostosis 1, craniosynostosis syndrome, Robinow Sorauf syndrome, Sweeney-Cox syndrome, Saethre-Chotzen syndrome
*WHSC1*	p.Ser4Asn	SIFT = 0.041 PhastCons = 1 GERP = 4.74	Autosomal dominant, sporadic mutation	Potentially significant variant from dedicated panel	4p partial monosomy syndrome
*MYOC*	p.Gln368^*^	PhastCons = 1 GERP = 4.52	Autosomal dominant	Known pathogenic variant beside dedicated panel	Primary open angle Glaucoma, primary Open angle glaucoma Juvenile onset 1

## Discussion

The above case report concerns a very rare clinical situation in pediatric oncology. To date, few cases of coexistence of various neoplasms in children have been described in the literature. Detailed statistical data defining the actual frequency of this phenomenon are missing. Due to the very low prevalence, there are no original papers about specific treatment strategies. The sequence of treatment and further outcomes depend on the type of coexisting tumors and their aggressiveness. It is also worth emphasizing that due to the similar and often nonspecific symptoms of neoplastic disease in the pediatric population, synchronous tumors constitute a diagnostic problem even for an experienced physician.

As mentioned above, only one case of the coexistence of HBL and NBL in a child has been already described (4). In both cases, the patients are infants and symptoms of the disease resulted from locally advanced HBL. However, there are some differences between the previously published case and the one being described in this report. In the previously reported case, multiple cutaneous capillary hemangiomas were found. In our patient, the presence of other tumors was excluded on the basis of a comprehensive physical examination and imaging tests. Furthermore, the patient presented in the current report was conceived naturally, while the previously described patient was born following an *in vitro* fertilization-assisted pregnancy. The treatment outcomes of both patients are also different. The infant described by Ozawa *et al*. died immediately after admission due to a ruptured liver which was caused by the developing HBL. In turn, our patient despite of the advanced initial stage of disease is 6 months after completion of treatment and remains in close follow-up. In both cases different genetic techniques were used and various results of the analyzes were also obtained. Ozawa *et al*. performed their testing on the material from the twin brother of the deceased patient and showed only Gaucher disease carrier. In our case, an advanced WES was carried out using NGS technology from material taken directly from the patient. Numerous potentially significant pathogenic variants were found (Table 1). However, there is a lack of genetic variants previously associated with Gaucher disease.

One of the main diagnostic problems in the our case was to determine the nature of the lesion in the left adrenal gland. The adrenal glands are one of the possible locations of distant metastases in HBL (7). This was indicated by the metastatic phase of the disease—the presence of multiple lesions in both lungs. However, several features typical for NBL, which suggested the presence of a second independent tumor already at the diagnostic stage may be noticed. The initial CT scan showed the presence of hypodense lesion with calcifications and no contrast enhancement. Numerous calcifications of adrenal gland tumor could suggest the diagnosis of NBL—they are present in about 79% of cases (8). The increase in their quantity was also documented at the time of tumor regression during chemotherapy (9). In most cases NBL manifests as a large heterogeneous tumor with focal necrosis or hemorrhage but small and accidentally discovered lesions may be homogeneous (10). The lack of a contrast enhancement is another feature that supports the diagnosis of NBL (11).

In our patient we also found the elevated values of normetanephrine and 3-methoxytyramine. The increase in the concentration of these methylated catecholamine metabolites is observed in most patients with NBL (12). An important issue was also the elevated NSE values in the perioperative period (Figure 2). NSE is a diagnostic marker in patients with NBL and its concentrations correlate with the stage of the disease (13).

Due to the local advancement and the presence of distant metastases, HBL was considered the main therapeutic target in the presented case. A satisfactory clinical, radiological and enzymatic response was observed during preoperative chemotherapy with SIOPEL-3 and SIOPEL-4 protocols dedicated to high and very high risk patients (5, 6). A simultaneous initial response of the NBL tumor and its subsequent stabilization were found. Two probable explanations for that situation may be proposed. First of all, NBL cells are sensitive to drugs used in HBL protocols—platinum derivatives and anthracyclines. They are present in chemotherapy regimens used in NBL treatment (14, 15). Secondly, the unique and frequently observed spontaneous regression among patients with NBL below 1 year of age cannot be excluded. This is also supported by the lack of *MYCN* amplification in our patient (16).

In the vast majority both tumors that occurred in our patient are sporadic. Genetic syndromes are associated with 15% of HBL while familial NBL occurs only in <5% of cases (17, 18). Several syndromes have been recognized as being associated with HBL and NBL. In our patient, whole-exome sequencing was carried out and revealed numerous cancer-predisposing genes. Mutations in *NF1, RAF1, TWIST1*, and *WHSC1* genes have been considered as cancer-related, therefore we decided to discuss their potential impact on patient's disease.

*NF1* (OMIM 613113) mutation in patients with NBL is a rare occurrence and, to our knowledge, has not been reported previously in children with HBL. Martinsson et al. (19) gave the first evidence of homozygous *NF1* deletion in NBL and suggested the role of *NF1* inactivation in the development or progression of this type of tumors. Origone et al. (20) reported *MYCN* amplification and chromosome 1p36 deletion in patient with *NF1* amplification and NBL. In presented case, an ultra-rare germline heterozygous missense mutation (p.Val2511Ile) in a highly conserved region (GERP >4.0 and PhastCons = 1) related with tumor predisposition has been reported.

NBL has been associated with several dominant disorders caused by germline mutations in RAS signaling pathway, involved in carcinogenesis. Noonan syndrome and LEOPARD syndrome (known as Noonan syndrome with multiple lentigines) are both related with germline *RAF1* mutations (OMIM 164760) and have been associated with increased risk of developing NBL (21). In our patient, potentially pathogenic non-synonymous variant in *RAF1* gene (p.Leu445Arg), not yet previously described in cancer-related conditions, has been found.

Of note, we revealed a novel germline insertion variant in *TWIST1* gene (p.Gly86dup) in patient with synchronous NBL and HBL. To date, *TWIST1* (OMIM 601622) has been associated with craniosynostosis 1, Robinow-Sorauf syndrome and Sweeney-Cox syndrome. The impact of *TWIST1* mutations on NBL has been rarely reported and remains unclear. Selmi et al. (22) showed upregulated *TWIST1* expression in *MYCN* amplified NBL cells. Additionally, *in vitro* studies found *TWIST1* to be a key regulator of the *MYCN* enhancer axis and a collaboration between *TWIST1* and *MYCN* may play role in NBL development (23).

NBL in a child with Wolf-Hirschhorn syndrome, caused by mutation in *WHSC1* gene (OMIM 602952), is a rare occurrence with only one case available in the medical literature (24). Our patient presented a potentially pathogenic mutation in *WHSC1* (p.Ser4Asn) gene with high SIFT deleteriousness score (<0.11) and high evolutionary conservation (GERP >4.7 and PhastCons = 1).

WES also detected known pathogenic variant in *MYOC* (p.Gln368^*^) reported as pathogenic with autosomal dominant inheritance for primary open angle glaucoma.

The issue of follow-up and prognosis in this type of patients remains unresolved. Complex genetic background has already led to the development of two independent tumors in the infancy. Undoubtedly, such unique genetic disorders may predispose to the further neoplasms in the future (25). In addition, the applied aggressive oncological treatment may also cause secondary cancers. Furthermore, extended genetic testing should also be carried out among the family members.

## Concluding Remarks

The coexistence of HBL and NBL represents extremely rare clinical issue in pediatric oncology. To our knowledge, presented case report is the second such description in the literature. The use of combined oncological treatment in our patient led to remission of both independent tumors. Based on performed testing, a specific signature of rare germline mutations can be proposed as a novel predisposing factor to synchronous HBL and NBL occurrence.

## Ethics Statement

Written informed consent was obtained from the patient's parents for the treatment according therapeutic protocols, genetic testing as well as for the publication of the case report and potentially-identifying information and images.

## Author Contributions

SS, KS, MaK, and WB contributed to case report concept and design. SS, KS, MaK, MiK, and MP wrote sections of the manuscript. TL, MH-K, MiK, MP, KK, LW, KW, and WG performed diagnostic tests and collected relevant clinical data. SS and WB critically revised the article. All authors were responsible for the integrity and accuracy of the data and approved the submitted version.

### Conflict of Interest Statement

Authors MiK, MP and KK are employed by company ‘Intelliseq sp. z o.o.’. The remaining authors declare that the research was conducted in the absence of any commercial or financial relationships that could be construed as a potential conflict of interest.
